# Time to sputum smear and culture conversions in multidrug resistant tuberculosis at University of Gondar Hospital, Northwest Ethiopia

**DOI:** 10.1371/journal.pone.0198080

**Published:** 2018-06-26

**Authors:** Agumas Shibabaw, Baye Gelaw, Shu-Hua Wang, Belay Tessema

**Affiliations:** 1 Department of Medical Microbiology, School of Biomedical and Laboratory Sciences, College of Medicine and Health Sciences, University of Gondar, Gondar, Ethiopia; 2 Department of Internal Medicine, Division of Infectious Diseases, The Ohio State University, Columbus, Ohio, United States of America; Fundació Institut d’Investigació en Ciències de la Salut Germans Trias i Pujol, Universitat Autònoma de Barcelona, SPAIN

## Abstract

**Background:**

Sputum smear and culture conversions are an important indicator of treatment efficacy and help to determine treatment duration in multidrug resistant tuberculosis (MDR-TB) patients. There are no published studies of sputum smear and culture conversion of MDR-TB patients in Ethiopia. The objective of this study is to evaluate and compare time to initial sputum smear and culture conversion and to identify factors influencing time to culture conversion.

**Methods:**

A retrospective cohort study was conducted among all culture positive and rifampicin mono resistant (RR) or MDR-TB patients from September 2011 to August 2016 at University of Gondar Hospital. Sputum cultures were collected monthly and conversion was defined as two consecutive negative cultures taken at least 30 days apart. Data were entered using EpiData and exported to SPSS software for analysis. Cox proportional hazard model was used to determine the predictor variables for culture conversion.

**Results:**

Overall, 85.5% (201/235) of the patients converted their cultures in a median of 72 days (inter-quartile range: 44–123). More than half (61.7%) of patients achieved culture conversion within three months. The median time for sputum smear conversion was 54 days (inter-quartile range: 31–72). The median time to culture conversion among HIV positive patients was significantly shorter at 67 days (95% CI, 55.4–78.6) compared to HIV negative patients, 77 days (95% CI, 63.9–90, p = 0.005). Independent predictors of significantly longer time to sputum culture conversion were underweight (aHR = 0.71, 95% CI, 0.52–0.97), HIV negative (aHR = 0.66, 95% CI, 0.47–0.94) and treatment regimen composition (aHR = 0.57, 95% CI, 0.37–0.88). Significantly higher rate of culture conversion was observed in 2015 (aHR = 1.86, 95% CI, 1.1–3.14) and in 2016 (aHR = 3.7, 95% CI, 1.88–7.35) years of treatment compared to 2011.

**Conclusions:**

Majority of patients achieved sputum culture conversion within three months and smear conversion within two months. Patients with identified risk factors were associated with delayed culture conversion. These factors should be considered during management of MDR-TB patients.

## Introduction

Sputum smear and culture conversion from positive to negative is one of the most important interim indicators of the efficacy of anti-tuberculosis treatment regimen. Sputum culture conversion is often used by clinicians to determine the duration of injectable agents and overall duration of multi-drug resistant tuberculosis (MDR-TB) treatment. Predicting the initial time to culture conversion is also important for planning and implementing respiratory isolation [1].

MDR-TB disease is a global challenge for TB programs. Ethiopia is among the 30 high burden TB, TB/HIV and MDR-TB countries that accounts for 80% of all new TB cases worldwide. In Ethiopia, the annual TB incidence is 192 per 100,000 population and the death rate is 26 per 100,000 population [2]. According to the 2014 Ethiopian National Drug Resistant TB Sentinel Report, the prevalence of MDR-TB was 2.3% and 17.8% among new and previously treated TB cases respectively and points toward an increasing trends in drugs resistant TB burden in the country [3].

The Ethiopian Federal Ministry of Health revised the National TB Control Program (NTCP) and introduced the guidelines on Programmatic Management of Drug Resistant Tuberculosis (PMDT) along with increasing medical and social services available for the management of MDR-TB patients. New recommendations reported for the use of sputum culture conversion status as a proxy marker of final treatment outcome [4–7] and also for determining the duration of treatment for MDR-TB patients. During the intensive phase, the PMDT recommends an injectable agent be continued for at least four months after the culture conversion or for at least eight months, whichever is longer [8]. The total duration of MDR-TB treatment should be continued for a minimum of 20 months or at least 18 months after the culture conversion patients are treated with a standardized treatment regimen for duration of 20–24 months or longer [8]. The duration of time that an MDR-TB patient remains infectious after the initiation of effective treatment is unclear. Delay in sputum conversion time may increase the likelihood that the patient will continue to be infectious and transmit *Mycobacterium tuberculosis* (MTB) to other individuals in his/her social network and the community [9]. TB programs have commonly recommended respiratory isolation and/or separation of MDR-TB patients on treatment until sputum smear or culture conversion has occurred which can take two to six months [10].

Different factors influencing the rate of sputum culture conversion among MDR-TB patients have been reported from different countries [5–7,9,11–13]. For example, anti-TB drug resistance pattern [14] and previous treatment with second line drugs (SLDs) [6] are all associated with increase delay in culture conversion. To the best of our knowledge, no prior study has been published on the time to culture conversion and factors associated with delay in culture conversion in Ethiopia. The aim of this study is to evaluate the rates of sputum smear and culture conversion and to identify predictors of sputum culture conversion in pulmonary MDR-TB patients in Ethiopia.

## Materials and methods

### Study design and settings

A retrospective cohort study was conducted in all pulmonary RR or MDR TB patients’ ≥ 15 years of age with positive baseline cultures and started SLD treatment at the University of Gondar Hospital from September 2011 to August 2016. The University of Gondar Hospital is located in Gondar City in Northwest Ethiopia and serves as a referral hospital for the region. A wide range of TB services including MDR TB inpatient ward and outpatient care center is present. Monthly sputum smear and culture for MDR TB patients in the region are collected and processed either at the Amhara Public Health Institute or at the University of Gondar Hospital TB culture laboratory. MDR-TB is defined as resistance to isoniazid (INH) and rifampicin (RIF) by phenotypic drug susceptibility test (DST) or genotypic drug resistance identified by a line probe assay (GenoType MTBDRplus V.2.0, HAIN Life Science, Nehren, Germany). Patients are diagnosed with presumed MDR TB if rifampicin mono resistance is detected on the Gene-Xpert assay (Cepheid, Sunnyvale, CA). Patients are also classified as presumed MDR TB if the patient has a history of prior TB treatment failure despite Directly Observed Therapy (DOT) or were close contacts of a patient with MDR-TB. Presumed MDR-TB patients were patients with MDR TB who were eligible for treatment and also included in the study. Patients were excluded from the study if they had negative cultures at baseline or cultures obtained at second or third months of follow up were contaminated or not collected.

### Treatment of multi-drug resistance TB

The standard University of Gondar Hospital SLD regimen for MDR TB contained at least three presumed to susceptible oral agents and an injectable agent. Oral agents included pyrazinamide (Z), Levofloxacin (Lfx), Ethionamide (Eto), Protonamide (Pto), Cycloserine (Cx) or Para-aminosalicyclic acid (PAS). Injectable agents were either an aminoglycoside (Amikacin [Am] or Kanamycin [Km]) or a polypeptide (Capreomycin [Cm]). Most patients were hospitalized at the initiation of therapy in accordance with national standards until sputum smear and/ or culture converted and was clinically stable. After discharge from the hospital, the patients were transferred to the treatment follow up centers (TFC) where daily DOTs were done. The patients returned to the University of Gondar Hospital Outpatient TB clinic monthly for follow up evaluation. Sputum smears and cultures were collected and processed monthly for all inpatients and outpatients [8].

### Outcome definitions

Sputum culture conversion was defined as the two consecutive negative cultures separated by at least 30 days. The documented date of sputum culture conversion is the date of the first consecutive negative sputum culture [15]. A positive culture was defined as ≥ one colony of *M*. *tuberculosis* [16]. Microscopy for positive acid-fast bacilli (AFB) was defined as positive smear with ≥ one AFB per 100 high-power fields (HPF) [16]. Baseline tests are any tests performed prior to starting SLD for MDR TB treatment.

### Data collection and statistical analysis

A standardized data extraction form was used to collect demographic, clinical, laboratory and microbiological data from their charts/cards, as well as treatment regimen from University of Gondar Hospital from November 2016 to March 2017. Demographic data included sex, age, weight, height, area of residence, smoking, alcohol use and chat chewing status. Clinical data include medical co-morbidities, history of TB exposure, previous TB treatment, previous treatment regimen composition including any SLDs and years of treatment started. Microbiological data included baseline and follow up sputum AFB smear and culture and DST results. In addition, baseline electrolytes, liver function tests and creatinine were also collected.

The data and laboratory results were double entered and cleaned in EpiData (v.3.1) software and exported to SPSS software package (version 20, Chicago, IL, USA) for statistical analysis. Proportions were computed for categorical variables and expressed as percentages, means, medians and inter-quartile ranges (IQRs). Time to initial sputum culture conversion was analyzed using the Kaplan-Meier method stratified HIV status, and differences in survival times across strata were assessed with the log-rank test. Patients were censored if sputum cultures did not convert before the last follow up. Univariate and multivariate Cox proportional hazards regression was used to identify independent predictors of time to initial sputum culture conversion. Backward selection was used to arrive at the final model. Hazard ratios (HRs) with 95% confidence intervals (CIs) were estimated for the effect of each variable on the initial culture conversion. Confounding and interaction were assessed by multivariable models. *P* ⩽ 0.05 was considered statistically significant.

### Ethical approval

The study was approved by University of Gondar Ethical Review Board (IRB) and permission letter was obtained from University of Gondar Referral Hospital. IRB waived the need for consent of each participant for their medical records to be used in this study. All collected data were kept confidential.

## Results

### Demographic and clinical characteristics

A total of 250 patients with either confirmed or presumptive pulmonary MDR-TB were started on MDR TB treatment regimen during the study period. Fifteen patients were excluded from the analysis due to negative culture at baseline or cultures obtained at second or third months of follow up were contaminated or not collected. A total of 235 patients met the inclusion criteria and were included in the final analysis. Of the 235 patients, 137 (58.3%) were confirmed to be MDR-TB by Geno Type MDRTB*plus*^®*l*^ ver 2 or phenotyping DST, 95 (40.4%) were rifampicin resistant by GeneXpert and 3 (1.3%) were presumptive MDR TB patients. Some patients were delayed to come to the treatment centers after diagnosed for MDR-TB which leads to delay in treatment imitation. The median age was 30 years (range 16 to 73 years, IQR = 23–40). One hundred forty two (60.4%) patients were male. The median body mass index was 17.1 (range from 9.65 to 24.99 kg/m^2^, IQR = 15.3–19.2). Among 34 patients whose sputum culture did not convert: Nineteen died, thirteen did not complete treatment and two were treatment failures.

HIV test was performed on 223/235 (95%) MDR-TB patients: 61 (26%) were HIV positive and 162 (68.9%) were HIV negative. Of the 61 HIV positive patients, 54 (88.5%) patients were on antiretroviral therapy (ART) before MDR-TB treatment initiation and 4 (6.5%) patients were on ART after MDR TB treatment initiation. Forty-four (18.5%) patients had current or previous smoking history, 17 (7.2%) had history of alcohol use and 13 (5.5%) chewed chat. Majority of the MDR-TB patients (64.3%) had BMI less than 18.5. Baseline data on liver function tests showed that 3.8% and 12.3% had abnormal level of ALT and AST, respectively. Baseline assessment on electrolyte level demonstrated that 12.8% and 10.2% of patients had low level of K+ and Na+, respectively. The level of creatinine was normal among 68.5% of patients. The mortality rate of patients within six month of MDR TB treatment initiation was 9.4%.

Two hundred fifteen (91.5%) of the MDR-TB patients were previously treated with first line drugs (FLDs). From 235 *M*. *tuberculosis* isolates, 137 isolates were resistant to both RIF and INH and 95 isolates were RIF mono-resistant. The treatment success rate of MDR TB patients was 72.2% (Table 1). Resistance to other first line drugs: 93.8% (30/32) ethambutol resistance, 12.5% (1/8) pyrazinamide resistance and 92.6% (25/27) streptomycin resistance.

**Table 1:** Demographic, clinical and microbiological characteristics of multi-drug resistant tuberculosis patients.

Characteristics	N (%)	Characteristics	N (%)
**Age (yrs)**		**Patient category**	
15–35	162 (68.9)	RR TB	95 (40.4)
36–55	65 (27.7)	MDR TB	137 (58.3)
>55	8 (3.4)	Presumptive MDR TB	3 (1.3)
**Sex**		**Previous history of TB**	
Female	93 (39.6)	New MDR case	20 (8.5)
Male	142 (60.4)	Previously treated with FLDs only (including STM)	215 (91.5)
**Residence**		**Resistance to RIF**	
Urban	132 (56.2)	Yes	232 (98.7)
Rural	103 (43.8)	Unknown	3 (1.3)
**Weight (kg)**		**Resistance to INH**	
< 40	52 (22.1)	Yes	137 (58.3)
≥ 40	172 (73.2)	Unknown	98 (41.7)
Missing	11 (4.6)		
**Alcohol use**		**ALT level (U/L) at baseline**	
Yes	44 (18.7)	Normal (7–56)	207 (88.1)
No	218 (81.3)	Abnormal (>56)	9 (3.8)
		Missing	19 (8.1)
**Smoking habit**		**AST level (U/L) at baseline**	
Yes	17 (7.2)	Normal (10–40)	189 (80.4)
No	218 (92.8)	Abnormal (>40)	29 (12.3)
		Missing	17 (7.2)
**Chat chewing habit**		**6 month Mortality (rate)**	
Yes	13 (5.5)	Yes	22 (9.4)
No	222 (94.5)	No	213 (90.6)
**BMI category**		**K+ level (mmol/L)**	
<18.5	151 (64.3)	Normal (3.5–5)	162 (68.9)
18.5–24.9	73 (31.1)	Low (<3.5)	30 (12.8)
Missing	11 (4.6)	High (>5)	5 (2.1)
		Missing	38 (16.2)
**HIV status**		**Na+ level (mmol/L)**	
Positive	61 (26)	Normal	53 (22.6)
Negative	162 (68.9)	Low	24 (10.2)
Unknown	12 (5.1)	High	4 (1.7)
		Missing	154 (65.5)
**ART initiation**		**Creatinine level (mg/dl, sex)**	
ART before MDR TB therapy	54 (88.5)	Normal (0.6–1.2 or 0.5–1.1)	161 (68.5)
ART while MDR TB therapy	2 (3.3)	Low (<0.6 or <0.5)	48 (20.4)
ART after MDR TB therapy	4 (6.6)	High (>1.2 or >1.1)	11 (4.7)
No ART Initiation	1 (0.4)	Missing	15 (6.4)
**Co-morbidity other than HIV**		**Smear status at baseline**	
No co-morbidity	227 (96.6)	Negative	59 (25.1)
Diabetes	5 (2.1)	Scanty (1–9 AFB/HPF)	11 (4.7)
COPD	1 (0.4)	1+	70 (29.8)
Fungus infection	2 (0.9)	2+	46 (19.6)
		3+	49 (20.9)
**Treatment regimen composition**		**Treatment outcome (n = 194)**	
Z-Cm-Lfx-Pto (Eto)-Cs	181 (77)	Cured	126 (64.9)
Z-E-Cm-Lfx-Pto (Eto)-Cs	12 (5.1)	Completed	14 (7.2)
Z-E-Cm-Lfx-Pto/Eto	5 (2.1)	Died	27 (13.9)
Z-E-Km(Am)-Lfx-Eto-Cs	33 (14)	Failure	2 (1)
Others	4 (1.7)	Rx non-completion	25 (12.9)
**Years of treatment (Rx) started**		**Treatment success rate (n = 194)**	
2011	27 (11.5)	Successful	140 (72.2)
2012	24 (10.2)		
2013	68 (29.8)	Poor	54 (27.8)
2014	55 (23.4)		
2015	44 (18.7)		
2016	17 (7.2)		

BMI- Body mass index, Rx- Treatment, HIV- Human immunodeficiency virus, ART- Anti-retroviral therapy, COPD- Chronic obstructive pulmonary disease, FLD- First line drugs, MDR-TB- Multi-drug resistant tuberculosis, RR- Rifampicin mono-resistance, RIF- Rifampicin, INH- Isoniazid, E- Ethambutol, Z- Pyrazinamide, STM- Streptomycin, Cm- Capreomycin, Km-Kanamycin, Am-Amikacin, Eto-Ethionamide, Pto- prothionamide, Cs- Cycloserine, Lfx- Levofloxacin, AST- Aspartate aminotransferase, ALT- Alanine transaminase, U/L-Unit per litter, mg/dl- milligram per deciliter, mmol/L- Millimol per litter

### Sputum smear status and conversion time

From 235 patients, 176 (74.9%) had positive sputum smear microscopy results at baseline: 4.7% were scanty, 29.8% were grade 1+, 19.6% were grade 2+ and 20.9% were grade as 3+. Of 176 patients with sputum smear positive, 157 (89.2%) patients converted sputum smear within 54 median days (IQR: 31–72, range 8 to 428) and 19 (10.8%) patients did not convert their sputum smear to negative.

### Initial time to sputum culture conversion

**Fig 1:**
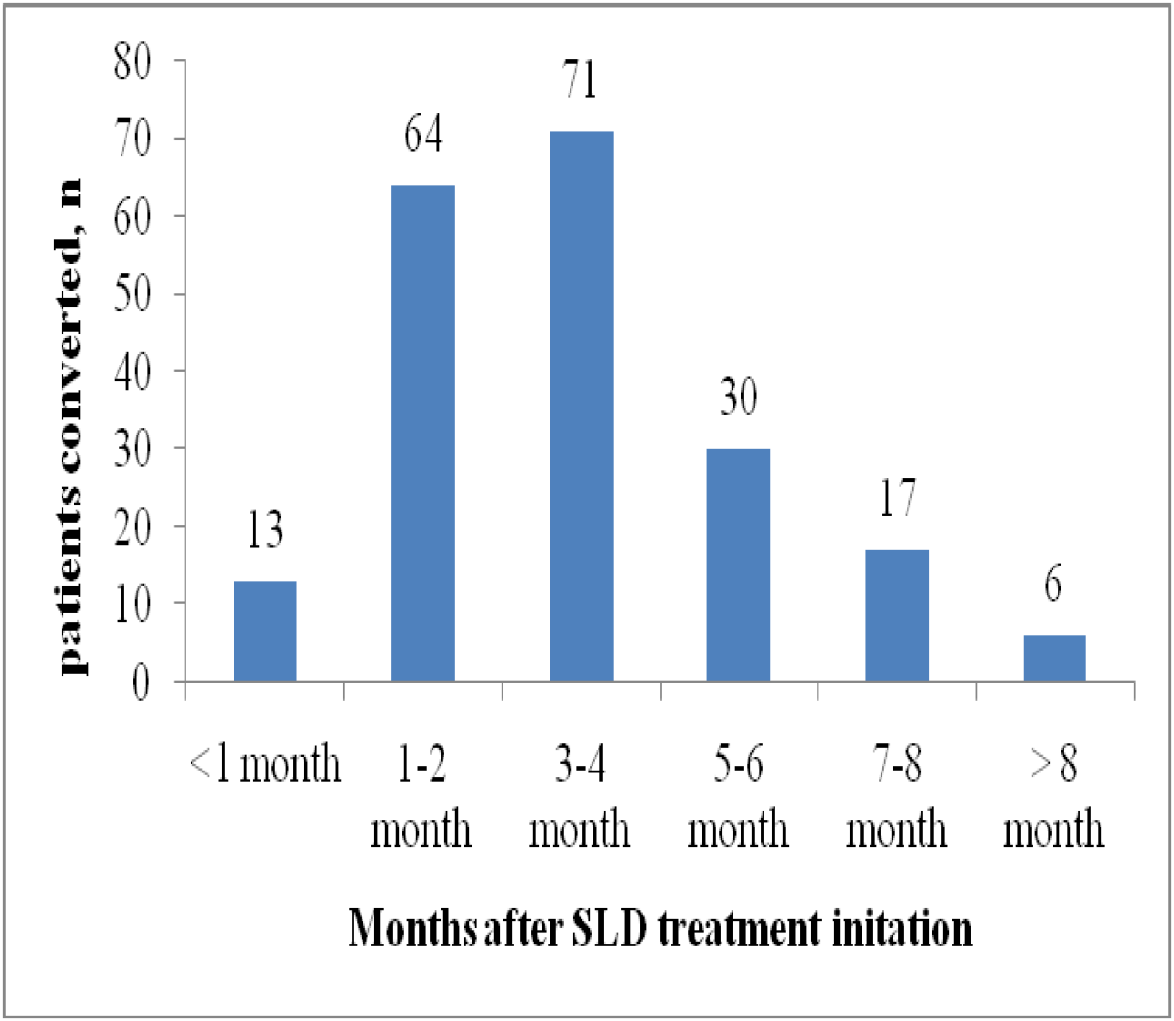
Initial sputum culture conversion in 201 of 235 culture-positive patients who had culture conversion.

Of 235 patients who were sputum culture positive at baseline, 201(85.5%) converted their culture in a median of 72 days (IQR, 44–123, range: 8 to 441 days, 95% CI, 64–79) and 34 (14.5%) patients did not have culture conversion. Seventy one (35.1%) patients were having initial sputum culture conversion within three to four months after SLD treatment initiation (Fig 1). Thirty-four (14.5%) of 235 patients with sputum culture positive at treatment initiation did not have culture conversion.

Although not statistically significant, the median time to culture conversion among those who were smear positive was 77 days (IQR, 50–123, 95% CI, 66–87) and 59 days for smear negative patients (IQR, 34–115, 95% CI, 43–70) (P> 0.05).

**Fig 2:**
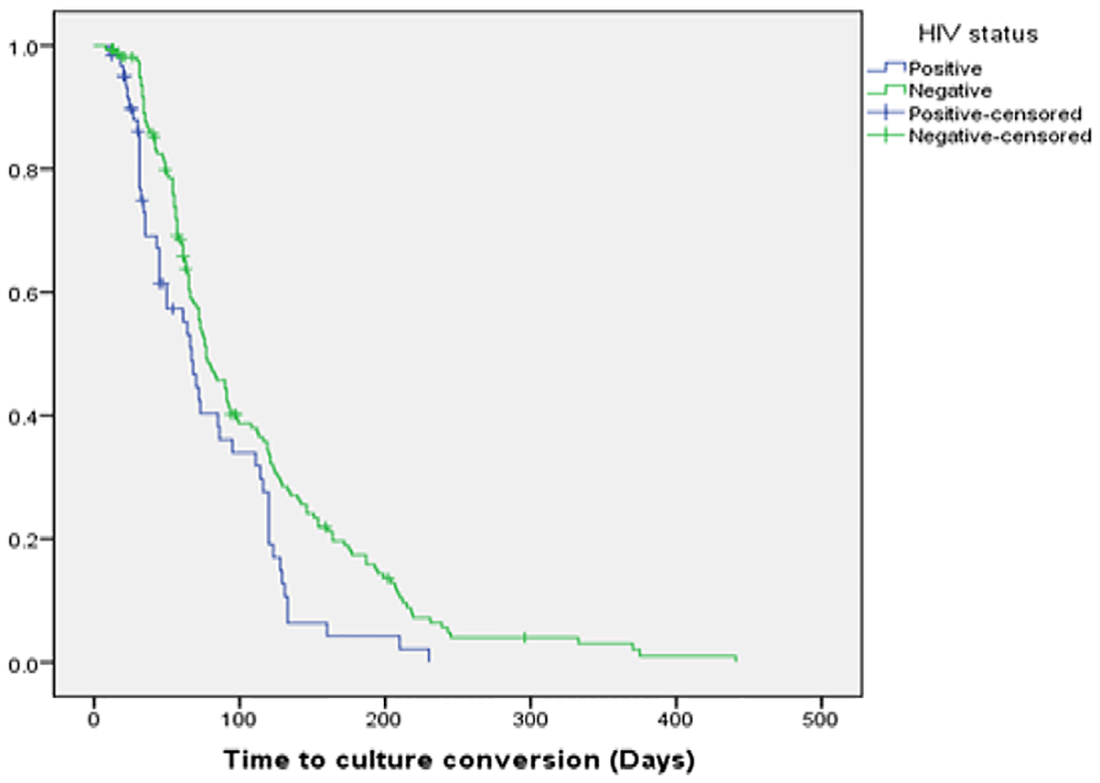
Kaplan-Meier survival plot of time to initial sputum culture conversion by HIV status.

There was a statistical difference between the median time to culture conversion of MDR TB patients who were HIV positive, 67 days (IQR, 30.5–112.5, 95% CI, 55.4–78.6) as compared to HIV-negative patients, 77 days (IQR, 49–128, 95% CI, 63.9–90, P = 0.005) (Fig 2).

### Predictors of time to initial sputum culture conversion

Univariate analysis showed that underweight, HIV negative, resistant to both RIF and INH drugs, previous history of TB, low creatinine level, treatment regimen composition (Z-E-Km (Am)-Lfx-Eto-Cs) and the year the treatment was started were all statistically significant predictors for delay in initial sputum culture conversion (Table 2).

**Table 2:** Univariate analysis of predictors of initial sputum culture conversion among multi-drug resistant tuberculosis patients*.

Characteristics	Number Converted (n = 201)	Converted (%)	cHR (95% CI)	P value
**Age (yrs)**				
15–35	142	87.7	Reference	
36–55	52	80	1.052 (0.67–1.44)	0.757
>55	7	87.5	0.77 (0.35–1.67)	0.514
**Sex**				
Female	82	88.2	Reference	
Male	119	83.8	0.86 (0.65–1.15)	0.32
**Residence**				
Urban	115	87.1	Reference	
Rural	86	83.5	1.02 (0.77–1.34)	0.91
**BMI category**				
<18.5 (Underweight)	127	84.1	0.71(0.52–0.96)	0.027
18.5–25 (Normal weight)	65	89.0	Reference	
**Alcohol abuse**				
Yes	34	77.3	1.08 (0.74–1.57)	0.66
No	167	87.4	Reference	
**Smoking habit**				
Yes	13	76.5	1.08 (0.61–1.90	0.78
No	188	86.2	Reference	
**Chat chewing habit**				
Yes	8	61.5	0.92 (0.45–1.87)	0.822
No	193	86.9	Reference	
**HIV status**				
Positive	50	81.9	Reference	
Negative	141	87	0.63 (0.45–0.86)	0.005
Unknown	10	83.3	0.77 (0.38–1.52)	0.45
**ART initiation**				
ART before MDR TB therapy	46	85.2	Reference	
ART after MDR TB therapy	3	75	0.9 (0.27–2.93)	0.86
No ART Initiation	1	1	0.58 (0.79–4.25)	0.59
**Patients category**				
RR TB	79	83.2	Reference	
MDR TB	120	87.6	0.63 (0.47–0.84)	0.002
Presumptive MDR TB	2	66.7	0.74 (0.18–3.02)	0.67
**Previous history of patients**				
New MDR case	17	85	Reference	
Previously treated with FLDs	184	85.6	0.68 (0.40–1.15)	0.15
**Co-morbidity other than HIV**				
No co-morbidity	196	86.3	Reference	
Diabetes	3	60	0.29 (0.04–2.07)	0.22
COPD	1	100	0.57 (0.06–5.52)	0.63
Fungus infection	1	50	2.98 (0.18–48.6)	0.44
**Baseline Smear status**				
Negative	44	74.6	Reference	
Scanty (1–9 AFB/HPF)	10	90.9	0.86 (0.43–1.73)	0.68
1+	63	90	0.96 (0.64–1.41)	0.83
2+	43	93.5	0.94 (0.61–1.44	0.78
3+	41	83.7	0.83 (0.54–1.28)	0.41
**Treatment outcome**				
Cured	126	100	Reference	
Completed	14	100	0.64 (0.44–0.91)	0.015
Died	8	29.6	0.39 (0.21–0.74)	0.004
Rx non-completion	12	48	0.00 (0.00)	0.944
Under treatment	41	100	0.45 (0.23–0.87)	0.018
**Baseline ALT level**				
Normal	177	85.5	Reference	
Abnormal	7	77.8	1.65 (0.77–3.55)	0.19
**Baseline AST level**				
Normal	162	85.7	Reference	
Abnormal	24	82.8	1.1 (0.71–1.69)	0.66
**Baseline Creatinine level**				
Normal	141	89.4	Reference	
Low	36	75	0.68 (0.47–0.99)	0.045
High	9	81.8	1.38 (0.70–2.72)	0.34
**Baseline Na+ level**				
Normal	49	92.5	Reference	
Low	21	87.5	1.13 (0.66–1.91)	0.64
High	4	100	1.81(0.64–5.08)	0.26
**Baseline K+ level**				
Normal	141	87	Reference	
Low	22	73.3	1.16 (0.74–1.82)	0.50
High	3	60	1.29 (0.4–4.07)	0.66
**Treatment regimen composition**				
Z-Cm-Lfx-Pto (Eto)-Cs	154	85.1	Reference	
Z-E-Cm-Lfx-Pto (Eto)-Cs	9	75	0.79 (0.4–1.55)	0.50
Z-E-Cm-Lfx-Pto/Eto	5	100	1.46 (0.59–3.58)	0.41
Z-E-Km(Am)-Lfx-Eto-Cs	29	87.9	0.55 (0.37–0.82)	0.004
Others	4	100	0.52 (0.19–1.42)	0.20
**Baseline resistance to INH**				
Yes	120	87.6	0.63 (0.47–0.84)	0.002
Unknown	81	82.7	Reference	
**Year of Rx started**				
2011	24	88.9	Reference	
2012	22	91.7	0.94 (0.52–1.69)	0.85
2013	60	88.2	1.78 (1.1–2.87)	0.017
2014	39	70.9	1.2 (0.72–2.00)	0.48
2015	39	88.6	1.71 (1.02–2.87)	0.041
2016	17	100	4.02 (2.13–7.58)	< 0.001

* Backward Cox regression was performed to calculate adjusted HR with variables that satisfied the criterion of P < 0.20 in the model.

BMI- Body mass index, Rx- Treatment, HIV- Human Immunodeficiency virus, ART- Anti-retroviral therapy, COPD- Chronic Obstructive Pulmonary Disease, FLDs- First line drugs, MDR-TB-Multi-drug resistant tuberculosis, RR- Rifampicin mono-resistance, INH- Isoniazid, E- Ethambutol, Z- Pyrazinamide, Cm- Capreomycin, Km-Kanamycin, Am-Amikacin, Eto-Ethionamide, Pto- prothionamide, Cs- Cycloserine, Lfx- Levofloxacin, AST- Aspartate aminotransferase, ALT- Alanine transaminase, cHR- Crude hazard ratio, CI- Confidence interval

In multivariable analysis, only underweight (aHR = 0.71 95% CI, 0.52–0.97, P = 0.029), HIV negative (aHR = 0.66, 95% CI, 0.47–0.94, P = 0.022) and usage of Z-E-Km (Am)-Lfx-Eto-Cs regimen (aHR = 0.57, 95% CI, 0.37–0.88, P = 0.011) were statistically significant predictors for longer time initial sputum culture conversion. Patients who started second line therapy in 2015 (aHR = 1.86, 95% CI, 1.1–3.14, p = 0.02) and 2016 (aHR = 3.7, 95% CI, 1.88–7.35, p = 0.001) have statistically significant higher rates of culture conversion as compared to those started in 2011 (Table 3).

**Table 3:** Multivariable analysis of predictors of initial sputum culture conversion among multi-drug resistant tuberculosis patients*.

Characteristics	aHR (95% CI) ^†^	P value
**BMI Category**		
<18.5 (Underweight)	0.71 (0.52–0.97)	0.029
18.5–25 (Normal weight)	Reference	
**HIV status**		
Positive	Reference	
Negative	0.66 (0.47–0.94)	0.022
Unknown	0.83 (0.39–1.78)	0.64
**Treatment regimen composition**		
Z-Cm-Lfx-Pto (Eto)-Cs	Reference	
Z-E-Cm-Lfx-Pto (Eto)-Cs	0.86 (0.43–1.70)	0.67
Z-E-Cm-Lfx-Pto/Eto	1.8 (0.74–4.63)	0.18
Z-E-Km(Am)-Lfx-Eto-Cs	0.57 (0.37–0.88)	0.011
Others	0.55 (0.20–1.52)	0.254
**Years of treatment initiation**		
2011	Reference	
2012	0.89 (0.49–1.60)	0.70
2013	1.62 (0.99–2.63)	0.051
2014	1.16 (0.68–2.96)	0.57
2015	1.86 (1.1–3.14)	0.02
2016	3.7 (1.88–7. 35)	0.001

^†^ HR <1 means that a patient with a risk factor has longer time to initial sputum culture conversion compared to a patient without this risk factor/ comparator.

*Only statistically significant results are given in the table.

aHR- Adjusted hazard ratio, CI- Confidence interval, BMI- Body Mass Index, HIV- Human Immunodeficiency virus, Z- Pyrazinamide, E- Ethambutol, Cm- Capreomycin, Km-Kanamycin, Am-Amikacin, Eto-Ethionamide, Pto- prothionamide, Cs- Cycloserine, Lfx- Levofloxacin

## Discussion

Prolonged periods of infectiousness increase the likelihood of spreading *M*. *tuberculosis* including MDR-TB [17]. Based on PMDT guideline of Ethiopia, at least one sputum sample for smear and culture should always be taken at initiation of MDR-TB treatment and repeat sputum specimen should be collected monthly [8]. Serial sputum smear and culture monitoring during MDR-TB therapy allows for assessment of sputum conversion which is important for clinical management and therapeutic planning. To the best of our knowledge, this is the first study in Ethiopia that evaluates time to sputum smear and culture conversion and predictors of initial sputum culture conversion among MDR-TB patients.

The present study revealed that 61.7% and 88.6% of MDR-TB patients achieved sputum culture conversion by the third and sixth months, respectively. This was comparable to the studies conducted in India (87%) [18], South Africa (89%) [11], Peru (92.9%) [12] and India (79%) [13] at the sixth month, and India (57%) [13] at the third month of treatment. However, other studies have reported relatively higher culture conversion rate by the third month in five countries (85%) [7] and in India (84%) [18] and by sixth month in India (98%) [19]. In our study, only 38.1% of patients had sputum culture conversion at the second month of treatment. This was comparable to findings conducted in Dominican Republic (48.8%) [20], Latvia (30%) [6] and Pakistan 53.4% [5] at the second month but lower than studies in India that reported a much higher percentage of sputum culture conversion at 2 months (58% to 82%) [18,19].

Since culture conversion is used as an early biomarker in treatment outcomes, the lower rates of initial culture conversion in our patients may be due to multiple factors for example unknown SLD susceptibility for most of our MDR TB patients. The comparable rates of culture conversion at the third and sixth months in our study are encouraging and indicate that the MDR-TB treatment and management at the study area is effective. Almost all of the patients were placed on second line treatment regimen after detection of RIF resistance by Gene-Xpert or line probe assays prior to SLD DST completion by standard methods. However, some delay in treatment initiation did occur when the patients failed to follow up at the MDR-TB treatment center after the diagnosis for MDR-TB. The presence of trained local health care workers and family treatment supporters for directly observed therapy (DOT) likely also played an important role in achieving good level of culture conversion at the third months of treatment when most of the patients were managed in the outpatient setting.

Two hundred one (85.5%) patients who were sputum culture positive at initiation of treatment, converted in a median of 72 days and 14.5% did not convert. The range of smear conversion time was lower than the range of culture conversion time as 51.6% of smear positive patients had low grade of bacilli load (+1 and scanty). In the present study, the median time to sputum culture conversion was less than previously reported in USA, 93 days [21], Delhi, 91.3 days [13], and London, 91 days [22] but more days than in Peru, 59 days [12], South Korea, 56 days [14], Indonesia, 60 days [23] and Dominican Republic, 60 days [20]. Achieving more rapid sputum culture conversion can simplify a patient’s therapy and increase comfort by reducing the amount of time she or he is given an intramuscular injectable drug and decrease auditory and vestibular toxicity associated with injectable agents. In addition, reducing the time to sputum culture conversion is an important infection control prevention measure because patients with positive sputum cultures are infectious and may transmit the disease to family members, health care providers and to the communities. This is especially true in limited resource settings, such as Ethiopia, where infection control prevention capacity is less adequate.

Delayed sputum culture conversion indicates poor treatment response. Knowing the risk factors associated with delayed culture conversion helps us to identify the patients that require more medical resources and attention, such as improve nutritional status, maintain prolonged respiratory isolation. In our study, underweight, being HIV negative and usage of Z-E-Km (Am)-Lfx-Eto-Cs regimen composition were independent predictors for low rate of sputum culture conversion in RR/MDR TB patients.

In our study, HIV-positive patients showed a statistically significant more rapid sputum culture conversion as compared to HIV negatives. We believe this is in part due to a fully integrated TB/HIV and MDR-TB/HIV care service delivery model throughout Ethiopia. This integrated HIV/MDR-TB model prioritized ART initiation, provided consistent follow-up and management of patients leading to improved treatment outcome. This finding is comparable with a study of HIV-positive patients in Peru associated with earlier culture conversion than HIV-negative patients [12]. But in contrast to our finding, a study in nine countries showed that HIV co-infected patients had significantly longer time to culture conversion as compared with HIV negative [4]. Moreover, other reports revealed that there is no difference in culture conversion rate based on HIV status [11,13,22,24]. This finding may be related to the pauci-bacillary nature of HIV and TB co-infection for early sputum conversion, low number of *M*. *tuberculosis* bacilli per milliliter of sputum in HIV positive patients [25]. Almost 85% of HIV positive patients from our study were started ART before MDR-TB therapy initiation and underlines the importance of integrating second-line TB therapy and ART to improve the MDR-TB cure rates in HIV co-infected patients.

BMI is the most useful tool to measure the level of malnutrition in adults, and low BMI (<18.5 kg/m^2^) is associated with increased TB related morbidity and mortality [26]. The mean BMI was 17.2 ± 3 at baseline and more than 64% of patients were considered malnourished with BMI < 18.5. Being underweight was significant predictor for longer time to sputum culture conversion as compared to normal weight. This finding is consistent with a previous studies from India [13], Indonesia [20], Georgia [27], South Korea [28] and a report from five low middle income countries [7]. The effects of malnutrition on decreasing immune function and leading to increase susceptibility to infectious diseases is well established [29]. A previous report documented that malnutrition reduced the concentrations of immunoglobulins, and CD4 and CD8 T-cells and natural killer cells among patients with TB [30]. This underscores the importance of close monitoring of patients with low BMI to improve nutrition and obtain adequate sputum collection to ensure culture conversion and implementing infection control measures to prevent disease transmission.

Composition of the MDR-TB treatment regimen influences the time to culture conversion [12]. Our results demonstrate that the recent recommended treatment regimen composition for MDR-TB treatment in Ethiopia (Z-Cm-Lfx-Pto (Eto)-Cs) was significant in decreasing days to culture conversion when compared to earlier MDR-TB regimen (Z-E-Km (Am)-Lfx-Eto-Cs). This finding might be due to the presence of large numbers of patients (86.3%) with unknown status of Ethambutol resistance in the previous regimen and all patients did not have DST results for SLDs that may contribute for longer time of culture conversion. Resistance to pyrazinamide or fluoroquinolones was an independent predictor for longer time of sputum conversion [7]. In addition to improved drug regimen, other advances in MDR-TB care recently in Ethiopia include improvement in the diagnosis with use of Gene-Xpert assay and early appropriate treatment initiation. This is consistent with our findings that showed patients who started therapy in recent years, in 2015 and 2016, had 2 and 4 times more rapid rate of sputum conversion, respectively as compared to those patients who started treatment in 2011. The Federal Ministry of Health in Ethiopia recently recommended that all MDR-TB isoalates should be tested for SLD resistance at baseline and all *M*. *tuberculosis* culture laboratory centers should perform DST for SLDs in the country [8]. This should further improve clinical outcome for MDR-TB patients by identify specific drug resistance and prescribing a more tailored MDR-TB drug regimen to patients in Ethiopia in the future. The notable limitation of the present study is its retrospective design, lack of DST for all SLDs and inability to evaluate culture re-conversion.

## Conclusions

Our finding shows that nearly 61% of MDR-TB patients achieve sputum culture conversion by the third month of treatment with median of 72 days. Underweight, HIV negative and the use of Z-E-Km (Am)-Lfx-Eto-Cs treatment regimen composition were associated with significant delayed culture conversion. Patients with lower BMI should be closely monitored during their MDR TB treatment course. Further studies are required to improve understand of the influences of sputum culture conversion on the treatment outcomes in drug-resistant TB patients.
